# Subepithelial Connective Tissue Grafting Around Dental Implants: From Established Soft-Tissue Benefits to Uncertain Marginal Bone Effects

**DOI:** 10.3390/jcm15176784

**Published:** 2026-09-01

**Authors:** Oskar Pieniak, Bartłomiej Górski

**Affiliations:** 1 Private Practice, 31-235 Kraków, Poland; 2 Department of Periodontology and Oral Mucosa Diseases, Medical University of Warsaw, 02-097 Warsaw, Poland

**Keywords:** subepithelial connective tissue graft, peri-implant phenotype, mucosal thickness, keratinized mucosa, supracrestal tissue height, marginal bone level, soft-tissue augmentation, dental implants

## Abstract

Subepithelial connective tissue grafting (SCTG) is widely used to increase peri-implant mucosal thickness and buccal soft-tissue volume, but its relevance to marginal bone stability remains uncertain. This narrative review examines whether SCTG provides clinically meaningful effects beyond soft-tissue phenotype modification, with particular emphasis on marginal bone level (MBL). Evidence was synthesized by separating direct intervention studies of SCTG or peri-implant soft-tissue augmentation from indirect studies relating baseline phenotype dimensions to subsequent tissue behavior. Randomized and comparative studies consistently show increases in mucosal thickness and soft-tissue volume and, in selected indications, improved contour and mucosal-margin stability. By contrast, MBL findings are heterogeneous. Greater mucosal thickness or supracrestal tissue height has been associated with less crestal remodeling, particularly during early healing; however, these prognostic associations do not establish that surgical thickening with SCTG reproduces a bone-preserving effect. Long-term trials further show that improved mucosal-margin stability can coexist with little or no clear between-group difference in MBL. Current evidence therefore supports SCTG primarily as a phenotype-modification and reconstructive procedure. A direct, independent, durable SCTG-mediated effect on marginal bone preservation remains biologically plausible but unproven; the evidence identified in this review does not support routine SCTG solely as prophylaxis for MBL preservation.

## 1. Introduction

SCTG is established for increasing peri-implant mucosal thickness and buccal soft-tissue volume, particularly at deficient or esthetically demanding sites. Whether this phenotype modification also improves adjacent hard-tissue stability remains uncertain.

The peri-implant phenotype includes MT, KMW, STH, and PBT. Greater MT or STH has been associated with more favorable crestal bone behavior, but these associations do not establish that surgical thickening with SCTG produces the same MBL effect [1,2,3,4,5,6].

Terminology is important when interpreting this literature. In this review, SCTG refers specifically to a subepithelial connective tissue graft used in a bilaminar soft-tissue augmentation procedure. The broader term connective tissue graft (CTG) is retained when it reflects the terminology of the cited study or when the harvesting technique is not sufficiently specified. A de-epithelialized gingival graft (DGG) is not considered synonymous with SCTG; studies using broader autogenous connective-tissue grafting are therefore interpreted according to the intervention actually reported.

This review separates prognostic phenotype evidence from intervention evidence. Its primary objective is to determine whether SCTG affects peri-implant tissue stability beyond soft-tissue thickening, with MBL as the central hard-tissue outcome, while identifying established reconstructive and esthetic indications.

## 2. Materials and Methods

### 2.1. Review Design and Scope

This work was designed as a focused narrative review rather than a systematic or scoping review. The synthesis was organized around a predefined clinical question: whether SCTG, beyond improving peri-implant soft-tissue dimensions, is supported by sufficient evidence for an additional effect on peri-implant tissue stability, particularly MBL. No PRISMA workflow, formal risk-of-bias instrument, or GRADE assessment was applied.

### 2.2. Literature Search

PubMed was used as the principal bibliographic database, with the original search updated through 12 August 2026. Reference lists of relevant systematic reviews, meta-analyses, consensus reports, randomized trials, and long-term follow-up studies were screened to identify additional publications, and targeted retrieval was used for later follow-up reports and key primary studies. During revision, a supplementary targeted literature update was performed in Scopus, Web of Science, and Embase using database-adapted combinations of the same core concepts (connective tissue grafting/soft-tissue augmentation, dental implants/peri-implant tissues, and marginal or crestal bone outcomes). This supplementary search was intended to broaden database coverage and identify potentially missed relevant studies rather than to convert the narrative review into a formally exhaustive systematic review. The core PubMed Boolean strategy combined intervention, phenotype, and bone terms: ((“connective tissue graft” OR “subepithelial connective tissue graft” OR SCTG OR CTG OR “soft tissue augmentation”) AND (implant OR “dental implant” OR “peri-implant”) AND (“mucosal thickness” OR “soft tissue thickness” OR “keratinized mucosa” OR “supracrestal tissue height” OR phenotype) AND (“marginal bone” OR “marginal bone level” OR “crestal bone” OR remodeling)). Additional targeted searches combined these concepts with immediate placement, delayed placement, augmentation timing, mucosal recession/dehiscence, esthetics, patient-reported outcomes, collagen matrices, and long-term follow-up. Searches were not restricted by publication year. Only English-language publications were included in the full-text evidence synthesis.

### 2.3. Evidence Selection and Prioritization

Studies were included when they addressed at least one component of the central framework: (i) SCTG/CTG or another defined peri-implant soft-tissue augmentation procedure with soft- or hard-tissue outcomes; (ii) MT, KMW, STH, or PBT in relation to MBL, mucosal-margin stability, or peri-implant health; or (iii) biological mechanisms required to interpret those clinical relationships. Publications not meeting these criteria were not used to support the principal treatment-effect conclusions. Systematic reviews/meta-analyses, randomized or controlled clinical trials, and long-term comparative studies were prioritized for treatment-effect claims; cohort studies were used mainly for prognostic associations; and histologic, consensus, translational, and preclinical evidence was used for biological context. The primary literature search and study selection were performed by O.P. The selected evidence and its interpretation were subsequently reviewed by B.G., who provided critical methodological and conceptual input during manuscript development. Follow-up publications were identified through citation tracking and targeted retrieval of the relevant trial families. When a primary study was also represented in a systematic review or meta-analysis, review-level evidence was used for broader context and the primary report for study-specific intervention, endpoint, and follow-up details; overlapping reports were not treated as independent evidence. Because this was a narrative review, no PRISMA-style excluded-study log was generated; publications outside the stated framework or the English-language full-text criterion were not used to support the principal treatment-effect conclusions.

### 2.4. Narrative Evidence Synthesis

**Figure 1:**
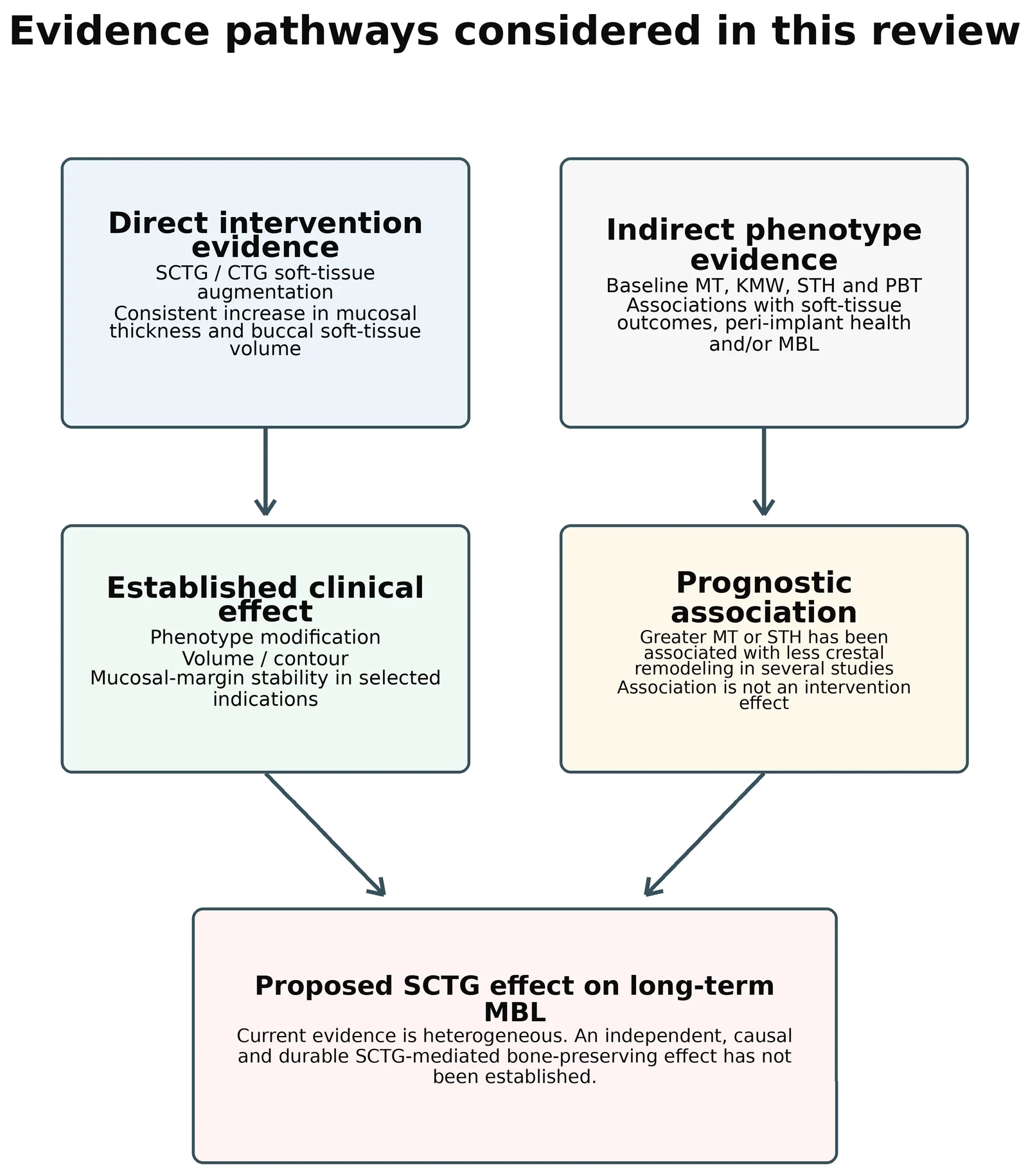
Evidence pathways linking SCTG, peri-implant soft-tissue phenotype, and marginal bone level (MBL). Direct evidence supports SCTG-related improvement in soft-tissue thickness/volume and related soft-tissue outcomes, whereas associations between phenotype dimensions and MBL provide indirect evidence. Current evidence does not establish a consistent, independent, long-term SCTG-mediated benefit on MBL. Representative direct soft-tissue augmentation evidence is summarized in [7], whereas representative phenotype–outcome evidence is summarized in [1,2,3,4,5,6].

**Figure 2:**
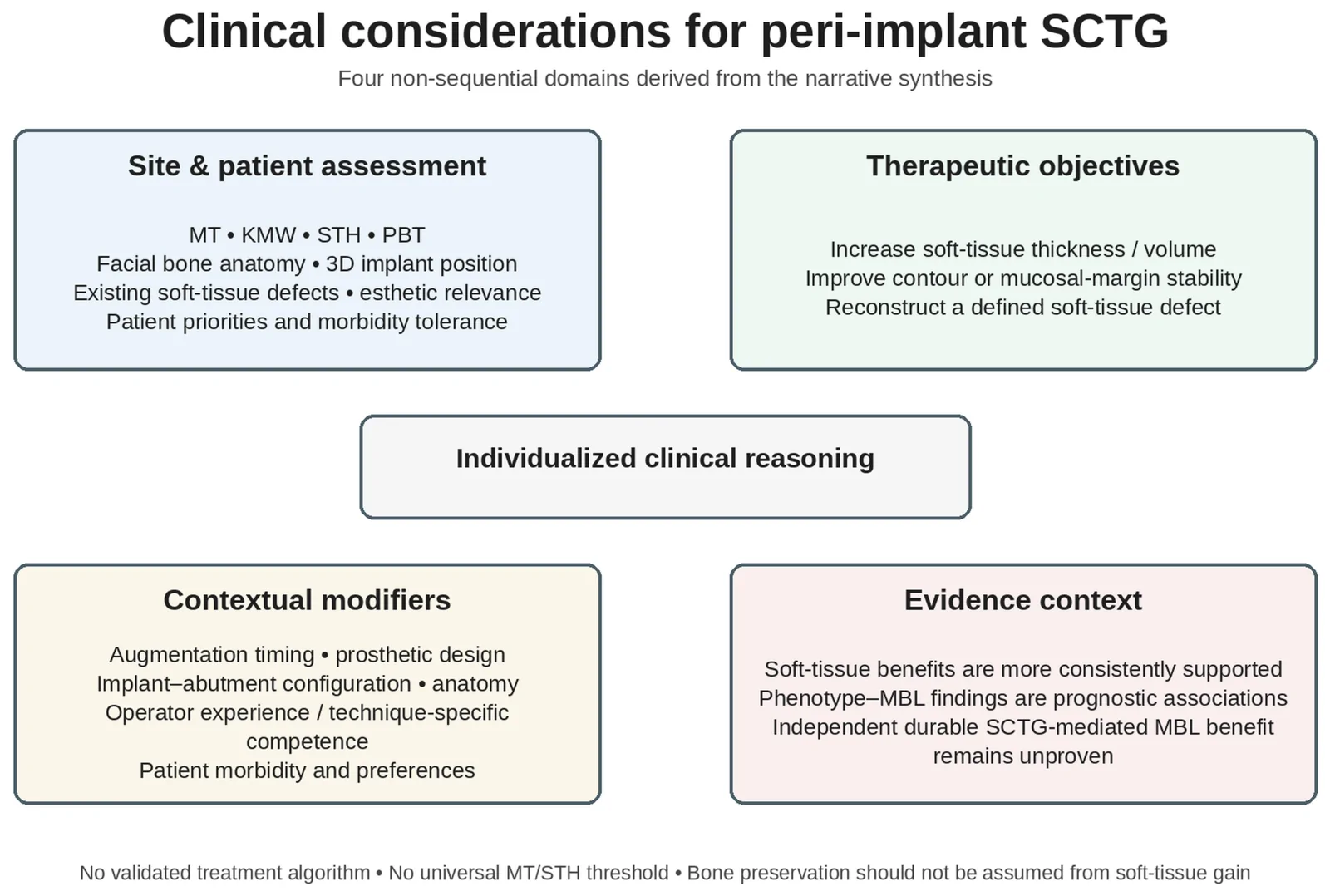
Clinical considerations for peri-implant SCTG derived from the narrative synthesis. The figure summarizes four non-sequential domains: site and patient assessment, therapeutic objectives, contextual modifiers, and evidence context. Operator experience/technique-specific competence is considered a contextual modifier rather than an indication for treatment. Phenotype–outcome associations should not be interpreted as treatment effects, and no universal MT or STH treatment threshold is proposed. The framework is not a validated treatment guideline.

Evidence was synthesized in three streams: direct intervention evidence (SCTG/CTG or soft-tissue augmentation with MBL measured as an outcome), indirect phenotype–outcome evidence (baseline MT/STH/KMW/PBT associated with subsequent outcomes), and contextual evidence (biology, esthetics, morbidity, and clinical indication). Claims concerning bone preservation were based preferentially on direct comparative evidence. Descriptions of the evidence in the tables are qualitative narrative summaries rather than formal certainty ratings; they reflect the design, directness, consistency, follow-up, endpoint relevance, sample size, and reported methodological limitations of the cited evidence without assigning GRADE-style categories. Generative AI (ChatGPT, OpenAI; GPT-5.6 Sol) was used only for English-language translation and linguistic editing and for preparation of Figures 1 and 2; it was not used for literature searching, study selection, evidence extraction, evidence synthesis, or scientific interpretation. All AI-assisted output was reviewed and verified by the authors.

## 3. Biological Basis

### 3.1. Peri-Implant Phenotype: Definition and Components

The peri-implant phenotype comprises mucosal thickness (MT), keratinized mucosa width (KMW), supracrestal tissue height (STH), and peri-implant bone thickness (PBT). These dimensions are related but not interchangeable; this review focuses on the soft-tissue components potentially modified by SCTG while treating PBT as an important co-determinant of outcome [7,8]. The evidence pathways are summarized in Figure 1.

MT is a horizontal mucosal dimension, KMW is the apico-coronal width from the mucosal margin to the mucogingival junction, and STH is the vertical distance between the alveolar crest and mucosal margin. Findings or thresholds for one parameter should therefore not be generalized to the others [7,8].

### 3.2. Soft-Tissue Barrier and the Implant–Tissue Interface

The peri-implant mucosa forms a specialized epithelial and connective-tissue barrier around the implant–abutment complex. Its organization differs from periodontal tissues around teeth and develops through healing and adaptation to the restorative components [9,10].

Histologic evidence supports the biological relevance of this barrier, but its clinical relationship with MBL requires separate evaluation.

### 3.3. Wound Healing, Revascularization, and Tissue Integration

Peri-implant healing involves inflammatory, proliferative, and remodeling phases. Revascularization and connective-tissue maturation explain how SCTG can integrate with the recipient bed and increase tissue dimensions [11,12].

Whether this integration also modifies marginal bone behavior is a separate treatment-effect question addressed by the clinical evidence below.

### 3.4. Crestal Bone Remodeling and Peri-Implant Endosseous Healing

Osseointegration, crestal remodeling, and soft-tissue healing occur concurrently. Early MBL may reflect physiological adaptation, whereas later changes can involve prosthetic, mechanical, inflammatory, and patient-related factors [13,14].

Implant position and depth, implant–abutment design, restorative geometry, buccal bone anatomy, plaque control, and patient factors can therefore modify the relationship between phenotype and MBL.

### 3.5. Supracrestal Tissue Height and the Soft-Tissue–Bone Interface

STH is the vertical soft-tissue compartment between the alveolar crest and mucosal margin and provides a conceptual link between soft tissues and crestal remodeling [8,9,15].

Studies of limited STH have reported greater early crestal remodeling, often using approximate 2–3 mm thresholds; however, protocols, implant depth, restorative components, and measurement methods vary [3,4,5,6,16,17,18,19].

These data support the relevance of STH but do not establish that SCTG predictably increases STH or that doing so produces durable MBL preservation.

### 3.6. The Proposed Soft-Tissue–Bone Relationship

Systematic reviews associate greater soft-tissue dimensions with more favorable MBL behavior, but effect magnitude varies with phenotype parameter, follow-up, implant configuration, and population [1,2,4].

This review therefore separates prognostic phenotype–MBL associations from intervention studies testing SCTG or other augmentation procedures.

### 3.7. Why SCTG May Modify the Peri-Implant Phenotype

SCTG can deliberately modify the peri-implant phenotype by increasing buccal MT and soft-tissue volume and improving contour [7,20,21,22].

Effects on KMW, STH, three-dimensional contour, and bone-related outcomes are distinct and technique-dependent; they should not be inferred from MT gain alone. The principal mechanisms and evidence pathways are summarized in Table 1.

MT is the phenotype component most directly targeted and most consistently increased by SCTG. KMW is a separate apico-coronal dimension and should not be assumed to increase simply because MT increases. STH is a distinct vertical supracrestal dimension; current evidence does not show that SCTG predictably increases STH or that an SCTG-related change in STH mediates durable MBL preservation [3,4,5,6,7,8,20,21,22].

## 4. Clinical Evidence

### 4.1. Soft-Tissue Effects of SCTG: Thickness, Volume, and Margin Stability

The most consistent clinical evidence concerns the direct soft-tissue effect of augmentation. Autogenous CTG/SCTG and selected collagen-based substitutes can increase peri-implant soft-tissue dimensions, with MT gain among the best-documented outcomes. The magnitude and durability of the gain depend on baseline phenotype, material, technique, timing, and the endpoint used [22,26,27,28,29,33,34,35,36,37].

In the 2026 multicenter trial by Cairo et al., both CTG and volume-stable cross-linked collagen matrix (VCMX) increased MT after 12 months, with a mean gain of 1.00 ± 0.75 mm for CTG and 0.66 ± 0.58 mm for VCMX; the adjusted between-group difference favored CTG by 0.37 mm. When baseline MT was ≥2 mm, the difference between treatments was smaller, suggesting that baseline tissue thickness may modify the relative benefit of autogenous grafting [26].

Soft-tissue augmentation is not fully characterized by a single linear thickness measurement. Profilometric studies have documented changes in three-dimensional buccal volume and contour, but MT, volume, and contour are distinct outcomes. Long-term comparative studies also show that early differences do not necessarily persist uniformly. Three-year data indicate stable tissues after both SCTG and VCMX in some settings, whereas 10-year follow-up suggests that selected collagen matrices may remain non-inferior for MT despite possible differences in contour behavior [22,27,28,30,37].

Across these studies, the most reproducible effect is greater peri-implant MT and, in appropriate settings, greater soft-tissue volume. The magnitude and persistence of this gain vary between protocols, but the soft-tissue effect itself is considerably more consistent than the reported changes in MBL.

### 4.2. Direct Intervention Evidence for Marginal Bone Level

Direct intervention studies measuring MBL after SCTG/CTG are central to the review; other augmentation modalities provide complementary evidence. Bone endpoints are heterogeneous—interproximal radiographic MBL, facial vertical level, buccal bone thickness, and 3D facial changes are not interchangeable.

In the pilot randomized clinical trial by Guglielmi et al., immediate implant placement with simultaneous CTG was compared with immediate implantation without grafting. At 6 months, CTG improved soft-tissue parameters, but significant between-group differences were not demonstrated for vertical buccal bone resorption or for most horizontal hard-tissue measurements. This illustrates that a clear soft-tissue effect does not necessarily produce a parallel difference across all hard-tissue endpoints [20].

The 5-year randomized trial by Zuiderveld et al. is particularly informative for the central question. CTG was associated with more favorable mid-buccal mucosal-level stability (approximately 0.1 mm versus −0.6 mm), whereas buccal bone thickness and MBL did not show clear between-group differences. This dissociation between a durable soft-tissue benefit and the absence of a parallel MBL advantage is one of the clearest examples of why mucosal-margin or MT improvement should not be used as a surrogate for bone preservation [24].

Comparative augmentation trials add further nuance. In Cairo et al., final 1-year MBL was similar after CTG and VCMX (approximately 0.47 and 0.43 mm, respectively), but because both groups received augmentation, the trial cannot determine whether augmentation is superior to no augmentation for MBL. Conversely, 3-year data from Surdiacourt et al. favored CTG over a collagen matrix for buccal soft-tissue profile and MBL in that specific comparison. This finding is clinically important but should not be generalized to CTG versus no augmentation or to all implant indications [26,30].

Thus, marginal-bone findings are less uniform than soft-tissue gains and vary with clinical model, comparator, implant/prosthetic configuration, endpoint, and follow-up.

### 4.3. Indirect Evidence: Soft-Tissue Phenotype and Marginal Bone Level

Baseline-phenotype studies add another layer to this interpretation. They help identify tissue configurations associated with different patterns of remodeling, although they address prognosis rather than the treatment effect of SCTG itself.

The 2023 meta-analysis by Bressan et al. included six randomized or controlled clinical trials and 354 implants with 10–14 months of follow-up. Implants with soft-tissue thickness ≥ 2 mm and <2 mm differed in marginal bone loss by approximately 0.54 mm, although heterogeneity was substantial (I^2^ > 50%). The result primarily concerns early remodeling and does not show what happens when a thin site is surgically thickened [1].

Tang et al. analyzed 14 studies and reported an overall MBL difference of 0.38 mm between thicker and thinner mucosal phenotypes. The association remained significant at ≤1 year (WMD 0.41 mm) but was not statistically significant at ≥3 years (WMD 0.17 mm; *p* = 0.07). This temporal pattern suggests that phenotype may be more strongly associated with early remodeling than with later MBL behavior [2].

The definition of phenotype is itself a limitation. A 2 mm threshold is widely used for MT, but measurement methods and anatomical reference points vary, and the peri-implant phenotype includes KMW, STH, and PBT as separate dimensions. Therefore, a finding for MT should not be generalized to the entire phenotype.

### 4.4. Does SCTG Preserve Marginal Bone? Critical Synthesis of the Evidence

Phenotype studies and augmentation trials address different questions: the former identify prognostic associations, whereas the latter test whether surgical modification changes outcome. A phenotype variable may therefore be prognostic without being a therapeutically modifiable determinant of MBL.

Intervention studies are heterogeneous. Some report favorable hard-tissue findings, whereas others show clear gains in MT, volume, or mucosal-margin stability without a corresponding MBL difference. Trials in which both groups receive augmentation inform material selection but cannot establish augmentation-versus-no-augmentation efficacy.

Overall, SCTG reliably increases peri-implant soft-tissue thickness and volume and can improve selected esthetic or margin outcomes. Bone findings are less consistent and depend on phenotype, implant timing and position, buccal bone anatomy, prosthetic configuration, comparator, and follow-up [1,2,3,4,5,6,20,24,26,30,38].

Long-term evidence supports the same distinction: Zuiderveld et al. found superior mucosal-margin stability without a clear MBL difference, while CTG-versus-matrix studies provide durability data but cannot establish superiority to no augmentation for bone preservation [24,28,30].

Recent randomized evidence further reinforces the separation between soft-tissue phenotype modification and hard-tissue preservation. In a 5-year randomized trial following alveolar ridge preservation, Zuiderveld et al. found no significant differences between CTG, xenogeneic collagen matrix, and no grafting for the primary mid-buccal mucosal-level outcome or secondary outcomes including marginal bone level [39]. In a separate three-arm randomized trial, Papantonatou et al. reported significantly greater soft-tissue thickness after SCTG or VCMX compared with no augmentation, whereas buccal bone thickness decreased over 6 months in all groups without significant between-treatment differences [40]. The latter study evaluated buccal bone thickness/remodeling rather than marginal bone level and should therefore be interpreted as evidence that successful soft-tissue thickening does not necessarily translate into preservation of the facial bone compartment.

### 4.5. Mucosal-Margin Stability, PSTD, and Peri-Implant Health

An important consequence of phenotype modification may be improved stability of the peri-implant mucosal margin, which is clinically distinct from MBL preservation. Cross-sectional and longitudinal data indicate that reduced buccal MT is associated with peri-implant soft-tissue dehiscence (PSTD), whereas adequate MT may contribute to long-term margin stability [31,32,41].

In a 5-year longitudinal cohort, Tavelli et al. identified MT measured 3 mm apical to the mucosal margin (MT3) as a strong predictor of margin stability; an MT3 threshold of approximately 2.23 mm was associated with complete stability in that cohort. The 2.23 mm value comes from a single cohort and requires external validation before broader clinical use [32].

The relationship between soft-tissue phenotype and peri-implant health is also clinically relevant. In the observational study by Obreja et al., sites/patients treated with soft-tissue volume grafting showed lower probing depth and bleeding on probing and a lower prevalence of peri-implantitis at follow-up (5.3% versus 13.9% at the patient level). Importantly, these findings should not be interpreted as evidence that soft-tissue grafting itself prevents peri-implantitis, because treatment allocation was not randomized for this endpoint and residual confounding cannot be excluded. KMW should be considered separately from MT; contemporary evidence associates adequate KMW with selected peri-implant health outcomes and, more consistently, with peri-implantitis risk than with mucositis incidence. Because these data are observational, they support risk associations rather than demonstrating that SCTG or surgical KMW augmentation prevents peri-implantitis [7,42].

Possible explanatory pathways include easier plaque control and less brushing discomfort where an adequate keratinized mucosal band is present, together with a more stable soft-tissue barrier and potentially lower local inflammatory burden. These mechanisms remain biologically plausible explanations rather than demonstrated mediators of a preventive SCTG effect; the observational evidence cannot establish that grafting itself caused the lower peri-implantitis prevalence [7,42].

### 4.6. Esthetic Outcomes, Patient-Reported Outcomes, and Morbidity

Esthetic outcomes depend on mucosal-margin position, buccal tissue volume, emergence contour, papillary fill, and tissue color. Soft-tissue augmentation is therefore particularly relevant when tissue deficiency or a high risk of recession/dehiscence is present. Nevertheless, improved MT does not guarantee a specific Pink Esthetic Score (PES) or patient-perceived esthetic benefit [33,43,44,45,46,47].

The 2025 meta-analysis by Ramanauskaite et al. found that autogenous soft-tissue grafting produced greater MT gain, while the difference in PES versus no augmentation was not statistically significant. Comparisons with collagen-based matrices sometimes favored autogenous grafts for professionally assessed esthetics, whereas patient-reported satisfaction was often similar. Three-year data from Jepsen et al. likewise showed high PES and patient satisfaction after both SCTG and VCMX [27,33].

Autogenous grafting also creates a donor site and may increase operative time and early treatment burden. In the Cairo multicenter RCT, CTG required approximately 10 additional minutes of chair time compared with VCMX, while postoperative pain and analgesic consumption were similar and the duration of discomfort was slightly shorter with VCMX. Ferrarotti et al. reported less early bleeding, pain, and swelling for VCMX in selected postoperative intervals. These differences emphasize that short-term morbidity and long-term satisfaction are separate outcome domains [26,44,48].

From the patient’s perspective, the expected soft-tissue benefit of SCTG should be weighed against donor-site morbidity, procedural burden, esthetic priorities, and individual preferences. Selected controlled studies and the broader evidence streams are summarized in Table 2 and Table 3.

## 5. Clinical Applications

### 5.1. Immediate Implant Placement and Simultaneous Soft-Tissue Augmentation

Immediate placement combines extraction-related remodeling, socket anatomy, and implant healing; baseline phenotype, facial bone anatomy, implant position, and anticipated contour changes are therefore particularly relevant [24,35,49,54].

Simultaneous SCTG may be considered for limited MT or buccal volume when esthetic demands are high because of site visibility and patient priorities or when contour change is anticipated. Controlled studies support soft-tissue volume and margin stability in selected cases; bone preservation should not be assumed [20,24,49].

SCTG does not compensate for incorrect three-dimensional implant position or replace hard-tissue reconstruction when the facial bone envelope is deficient. A thin phenotype combined with a thin or compromised buccal plate may represent a higher-risk tissue configuration, but treatment planning should address the underlying anatomical problem rather than rely on soft-tissue grafting alone.

### 5.2. Delayed Implant Placement and Soft-Tissue Augmentation

In delayed placement, early post-extraction remodeling has occurred and residual deficiencies can be assessed directly. SCTG may increase buccal volume, improve contour or margin stability, and reconstruct soft-tissue defects [33,35,50].

When healed tissues are favorable, added grafting may offer limited benefit; persistent volume deficiency, contour loss, or dehiscence provides a clearer reconstructive indication.

### 5.3. Simultaneous Versus Staged Augmentation

Timing should follow the therapeutic objective and anatomy. SCTG may be performed at implant placement, uncovering, or after prosthetic reconstruction [50].

Simultaneous augmentation modifies tissues during initial healing, whereas staging permits reassessment after stabilization. Current evidence does not establish one universally superior timing strategy.

### 5.4. Clinical Considerations Derived from the Narrative Synthesis

SCTG should not be indicated on the basis of a single measurement. MT, KMW, and STH are separate soft-tissue dimensions and should be considered together with PBT/buccal bone anatomy, three-dimensional implant position and timing, prosthetic design, existing defects, esthetic relevance, patient preferences and tolerance for procedural morbidity, and operator experience/technique-specific competence [3,8,18,25].

A thin phenotype combined with a thin or discontinuous facial bone plate may increase susceptibility to contour changes during healing. In contrast, when soft-tissue dimensions and volume are adequate, the buccal bone envelope is favorable, implant position is correct, and no specific reconstructive or esthetic indication is present, the evidence identified in this focused review does not currently support routine SCTG solely for MBL preservation. These factors should therefore be interpreted as clinical considerations arising from the narrative synthesis rather than as components of a prescriptive treatment algorithm. Figure 2 summarizes the principal assessment domains, therapeutic objectives, contextual modifiers, and evidence context relevant to peri-implant SCTG. The framework has not been prospectively validated and does not constitute a clinical practice guideline [25].

Esthetic relevance should be assessed multidimensionally rather than reduced to a binary low/high-risk label. Validated esthetic indices such as the Pink Esthetic Score can structure outcome assessment, but they do not by themselves constitute a preoperative risk classifier; three-dimensional implant position, soft-tissue phenotype and existing defects, visibility of the site, and patient expectations remain important contextual considerations [33,43,44,45,46,47].

Existing facial soft-tissue dehiscence/deficiency represents a different indication from prophylactic thickening. The classification proposed by Zucchelli et al. emphasizes the need to characterize the defect, peri-implant tissue levels, implant position, and associated anatomical conditions before selecting treatment. SCTG may be used to increase thickness and volume and, in selected cases, to obtain coverage of an exposed implant surface; however, predictability depends on defect characteristics and correction of the underlying cause [41,47].

Recent longitudinal data provide a more refined perspective on MT. In the esthetic zone, low buccal MT/MT3 may identify sites at greater risk of mucosal-margin instability. The cohort-derived MT3 value of 2.23 mm should not be interpreted as a universal surgical threshold or converted into a generalized treatment range; rather, it supports incorporating site-specific MT into risk assessment [31,32].

STH should likewise be assessed independently from MT. Values in the approximate ≤2–3 mm range have been associated with greater crestal remodeling in several studies, but the relevant values are protocol-dependent and should not be interpreted as universal treatment thresholds. Implant depth, implant–abutment configuration, restorative components, and measurement protocol should be considered. A strategy intended to increase the vertical supracrestal dimension may be considered in selected sites, but evidence for a specific SCTG → STH → long-term MBL causal pathway remains limited [3,4,5,6,17,18,19]. These phenotype- and context-dependent considerations are summarized in Table 4.

## 6. Future Research Priorities

Future trials should address the causal question directly: whether SCTG provides a long-term benefit for MBL compared with no soft-tissue augmentation after adequate characterization of baseline soft-tissue phenotype and hard-tissue anatomy. Interproximal radiographic MBL should be prespecified as a primary or key secondary endpoint when marginal bone preservation is the research question. Facial/buccal vertical bone-level change, buccal bone thickness or dimensional change, and three-dimensional hard-tissue outcomes should be reported separately rather than treated as interchangeable measures of MBL.

Prospective studies should standardize measurements of MT, KMW, STH, and PBT and report facial bone anatomy, three-dimensional implant position, augmentation timing, and defect morphology. Implant-related variables that could modify or confound observed marginal bone changes should also be documented and, where feasible, incorporated into stratified or adjusted analyses. These include implant design characteristics, implant–abutment configuration, and platform-switching versus platform-matching design. Prosthetic variables, including retention mode (cement- versus screw-retained), abutment material, and prosthetic emergence characteristics, should likewise be documented so that their potential contribution is not attributed to the soft-tissue intervention.

Direct SCTG-versus-no-soft-tissue-augmentation comparisons remain particularly important for determining whether grafting itself modifies hard-tissue outcomes. Trials comparing SCTG/CTG with alternative materials can address comparative effectiveness, morbidity, and material selection but should not substitute for a non-augmented control when the causal question concerns MBL preservation [35,38,52,53]. Publications representing sequential follow-up of the same randomized cohort should be identified as such so that long-term durability is distinguished from independent replication.

Stratification by baseline MT and STH should be prespecified where appropriate because treatment effects may differ according to baseline phenotype and may be diluted when sites with already favorable dimensions are included. However, currently reported MT and STH values should not be converted into universal eligibility or treatment thresholds without prospective validation.

Long-term follow-up should assess hard- and soft-tissue outcomes in parallel, including MBL, mucosal-margin stability, peri-implant health, esthetics, patient-reported outcome measures, and morbidity. Serial non-invasive assessment of peri-implant soft-tissue dimensions using intraoral ultrasonography is promising because it may facilitate repeated site-specific measurements of MT without tissue penetration. Future studies should establish measurement reproducibility, standardized acquisition protocols, and sensitivity to longitudinal tissue changes to determine its role in monitoring peri-implant soft-tissue dimensions [55].

## 7. Limitations of the Review

This narrative review was initially centered on PubMed, citation tracking, and targeted retrieval; during revision, supplementary targeted searches were additionally performed in Scopus, Web of Science, and Embase. These searches broadened database coverage but were not designed as a formally exhaustive systematic-search workflow. No formal risk-of-bias tool or GRADE framework was applied. Heterogeneity in definitions and measurement of MT, STH, KMW, PBT, and MBL, as well as implant, prosthetic, and augmentation protocols, limits direct comparison. CTG and SCTG terminology and harvesting methods are also inconsistent. The primary search and study selection were performed by one reviewer and critically reviewed by the second author, so selection bias cannot be excluded. The evidence summaries used in this narrative review are descriptive and should not be interpreted as formal certainty-of-evidence grades.

## 8. Conclusions

SCTG provides predictable benefits for peri-implant soft-tissue phenotype modification, particularly by increasing mucosal thickness and volume and, in selected indications, improving contour and mucosal-margin stability. However, associations between greater baseline MT or STH and favorable marginal bone behavior should not be interpreted as evidence that surgical phenotype modification produces the same effect. Current direct and long-term comparative evidence does not establish an independent, durable SCTG-mediated benefit for MBL preservation. Accordingly, the evidence identified in this focused review supports SCTG primarily for established soft-tissue, reconstructive, esthetic, and patient-centered objectives and does not currently support routine use solely to prevent marginal bone loss. Whether selected high-risk phenotypes may derive an additional bone-related benefit remains an important question for future randomized trials.

## Figures and Tables

**Table 1:** Biological mechanisms and evidence pathways linking the peri-implant phenotype with marginal bone stability.

Mechanism	Type of Evidence	Biological Rationale	Phenotype Relationship	Relevance to MBL	Interpretive Summary	References
Soft-tissue barrier and integration	Histologic and biological evidence	A specialized soft-tissue interface forms around implants and contributes to local homeostasis.	Tissue dimensions and quality may influence the local soft-tissue compartment.	Provides biological plausibility for a soft-tissue–bone relationship but does not prove SCTG-mediated bone protection.	Biological rationale is established; a causal SCTG → MBL effect is not demonstrated.	[7,9,10,15]
Healing and revascularization	Reviews and clinical translational data	Healing includes inflammatory, proliferative, and remodeling phases; vascularization supports graft integration.	SCTG may modify tissue volume and the healing environment.	Mechanistic support only; revascularization is not a surrogate endpoint for long-term MBL.	Mechanistic evidence supports graft integration; no direct long-term MBL inference.	[11,12]
Supracrestal tissue dimensions	Histologic, controlled clinical, and review evidence	The supracrestal compartment forms the biological interface between the bone crest and mucosal margin.	STH is a distinct vertical phenotype dimension.	May be relevant to early remodeling, but greater STH does not automatically prevent bone loss.	Associations with remodeling are reported, but findings are heterogeneous and protocol-dependent.	[3,4,5,6,15,16,17,18,19]
MT/soft-tissue phenotype ↔ marginal bone	Systematic reviews and meta-analyses	Thicker tissues may be associated with a different crestal bone response.	Directly concerns a phenotype component targeted by augmentation.	Supports an association, not a causal SCTG effect.	Association evidence is present; a causal SCTG-mediated MBL effect remains uncertain.	[1,2,23]
Implant position and buccal bone as modifiers	Systematic review/clinical evidence	Buccal bone dimensions, implant position, and prosthetic factors influence tissue remodeling.	These factors may modify or confound phenotype–MBL associations.	Essential when interpreting hard-tissue outcomes in SCTG studies.	Relevant contextual and modifying factors; not direct evidence of an SCTG treatment effect.	[24,25]
Phenotype modification with SCTG	Randomized clinical trials	SCTG increases soft-tissue dimensions and may modify contour/phenotype.	Direct intervention targeting a modifiable phenotype component.	Provides the intervention link but does not itself prove long-term MBL preservation.	Consistent direct evidence for soft-tissue gain; evidence for an independent MBL effect remains uncertain.	[20,22,26,27,28,29,30]
MT and mucosal-margin stability	Cross-sectional and longitudinal studies	Adequate buccal MT may increase resistance of the mucosal margin to apical displacement.	MT3 was a strong predictor of 5-year margin stability; the 2.23 mm threshold requires external validation.	Primarily relevant to PSTD/mucosal-margin stability; not direct SCTG → MBL evidence.	Prognostic association with mucosal-margin stability; intervention causality remains uncertain.	[31,32]

Note: The interpretive summaries in this table are qualitative narrative descriptions and are not formal certainty-of-evidence ratings or GRADE assessments.

**Table 2:** Selected randomized and controlled clinical studies evaluating SCTG or peri-implant soft-tissue augmentation.

Study (Ref.)	Sample Size	Context/Follow-Up	Intervention vs. Comparator	Soft-Tissue Outcome	MBL/Hard-Tissue Outcome	Esthetics/PROMs	Main Interpretation
Guglielmi et al. (2022) [20]	30 randomized; 26 at 6 months	Immediate placement; 6 months	SCTG vs. no SCTG	SCTG maintained contour and increased soft-tissue thickness/volume versus no SCTG.	No significant between-group difference in vertical buccal bone resorption; most horizontal buccal bone measurements were comparable, although coronal ridge changes favored SCTG.	—	Pilot RCT: clear soft-tissue benefit without a parallel difference across most hard-tissue endpoints.
Zuiderveld et al. (2024) [24]	60 randomized (30/group); 54 at 5 years	Immediate placement, esthetic zone; 5 years	SCTG vs. no SCTG	Less mid-buccal mucosal recession with SCTG (change 0.1 vs. −0.6 mm).	Buccal bone thickness and MBL were stable, with no clear between-group differences.	High esthetic scores and patient satisfaction in both groups; no clear between-group difference.	Long-term soft-tissue benefit did not translate into a parallel MBL advantage.
Ferrarotti et al. (2025) [48]	44 (22/group)	Small buccal dehiscence ≤3 mm; 12 months	CTG vs. VCMX	Comparable 3-month augmentation; at 1 year CTG showed greater profilometric stability (0.21 vs. −0.05 mm).	Radiographic bone-level change favored CTG at 1 year (0.09 vs. −0.34 mm); other radiographic measures were comparable.	Early bleeding, pain and swelling favored VCMX at selected time points.	Indication-specific CTG advantage at 1 year; not a CTG-versus-no-augmentation comparison.
Surdiacourt et al. (2025) [30]	60 randomized (30/group); 50 at 3 years	Single implants; 3 years	CTG vs. collagen matrix	Greater long-term buccal soft-tissue profile gain with CTG.	Less MBL with CTG in this specific material-to-material comparison.	Secondary clinical/esthetic outcomes showed limited differences.	Important CTG-vs-matrix comparison; not proof of universal CTG-mediated bone protection.
Hadzik & Dominiak (2026) [29]	36 patients; 40 sites (CTG 16, VCMX 16, control 8)	Esthetic-zone implant sites; 12 months	CTG vs. VCMX vs. no augmentation	Both augmentation groups had significantly greater STT than control; CTG tended to be 0.3–0.6 mm thicker than VCMX at 12 months.	MBL was low and comparable among groups.	No biological or technical complications reported.	Three-arm RCT supports a soft-tissue effect but not a short-term MBL advantage.
Cairo et al. (2026) [26]	100 randomized; 98 completed (49/group)	Implant sites; 12 months	CTG vs. VCMX	MT gain favored CTG (1.00 ± 0.75 vs. 0.66 ± 0.58 mm); difference diminished when baseline MT ≥ 2 mm.	MBL was not the primary endpoint; between-group bone findings do not establish augmentation-versus-no-augmentation efficacy.	VCMX shortened chair time by ~10 min and slightly reduced duration of discomfort; final satisfaction was similarly high.	Supports phenotype-dependent material selection; MT superiority should not be extrapolated to bone preservation.
Jepsen et al. (2026) [27]	56 analyzed (28/group) at 3 years; original RCT 88	Implant sites; 3 years	SCTG vs. VCMX	MT decreased over time in both groups; change did not differ significantly (estimated difference 0.2 mm, *p* = 0.587); recession was minimal.	Not a principal bone endpoint in this follow-up.	PES and patient satisfaction were high and similar; clinician overall esthetic rating favored SCTG.	Both treatments maintained stable soft tissues; no uniform long-term SCTG superiority across outcomes. This report is a follow-up of the original multicenter RCT and should not be counted as an independent trial family.
Strauss et al. (2026) [28]	20 randomized; 10 at 10 years (5/group)	Single implants; 10 years	SCTG vs. VCMX	VCMX was non-inferior to SCTG for MT; trend toward greater long-term contour reduction with VCMX.	Bone outcomes were secondary; small follow-up sample limits inference on MBL.	PES and OHIP-14 did not differ significantly.	Useful durability evidence, but attrition and secondary bone endpoints preclude strong MBL conclusions. This 10-year publication is a follow-up of the same 20-patient randomized cohort reported at 5 years by Thoma et al. [22]; it informs durability rather than independent replication.
Thoma et al. (2023) [22]	20 randomized	Post-loading implant sites; 5 years	SCTG vs. VCMX	Median MT change from baseline to 5 years was 0.3 mm in both groups; ridge-contour changes were also similar.	Clinical and radiographic peri-implant outcomes remained stable without significant intergroup differences.	Favorable esthetic and patient-centered outcomes in both groups.	Five-year material comparison supports similar stability, not a unique SCTG-mediated bone effect. This 5-year publication reports the same randomized cohort later followed to 10 years by Strauss et al. [28] and should therefore be interpreted as durability evidence rather than a separate independent replication.
Schmitt et al. (2021) [37]	14 (7/group)	Contour augmentation; 6 months	SCTG vs. porcine collagen matrix	At 6 months, volume and thickness gains numerically favored SCTG (61.75 vs. 19.56 mm^3^; 0.80 vs. 0.30 mm), without significant intergroup differences.	Not assessed as a long-term MBL endpoint.	—	Controlled early volumetric evidence; does not address long-term MBL preservation.
Zuiderveld et al. (2025) [39]	60 patients (CTG 20, XCM 20, no graft 20)	RCT; implant placement after alveolar ridge preservation; 5-year follow-up	CTG vs. XCM vs. no soft-tissue grafting	No significant between-group difference at 5 years for the primary mid-buccal mucosal-level outcome.	No significant between-group differences for secondary outcomes including marginal bone level.	—	Direct long-term augmentation-versus-no-augmentation evidence; no demonstrated CTG-specific MBL advantage.
Papantonatou et al. (2026) [40]	25 patients; three-arm randomized controlled trial	Simultaneous augmentation at implant placement; 6-month follow-up	SCTG vs. VCMX vs. no augmentation	SCTG and VCMX increased soft-tissue thickness versus control.	Buccal bone thickness decreased over time in all groups without significant between-treatment differences; MBL was not the assessed hard-tissue endpoint.	Patient-reported outcomes were assessed in the trial; they are not used here to support the MBL conclusion.	Supports dissociation between soft-tissue thickening and facial bone preservation; BBT/remodeling should not be conflated with MBL.

Note: Several publications in Table 2 are sequential follow-up reports from earlier randomized cohorts. They are retained to show durability of outcomes and should not be interpreted as independent replication when they belong to the same trial family. Intervention terminology follows the original reports: SCTG is used when the subepithelial bilaminar procedure is sufficiently specified, whereas CTG is retained when procedural detail does not permit more specific classification. Comparisons with a non-augmented control provide direct augmentation-versus-no-augmentation evidence; CTG/SCTG-versus-matrix trials primarily inform comparative material performance and are not treated as evidence that augmentation itself preserves MBL. Sample size is reported as randomized and, where available, analyzed at the stated follow-up; endpoint terminology is retained according to the original study and is not assumed to be interchangeable.

**Table 3:** Evidence streams from systematic reviews, meta-analyses, and long-term syntheses.

Evidence Stream	Design/Scope	Main Question	Synthesis	Direct SCTG → MBL Evidence?	Interpretive Limitation	Ref.
Soft-tissue thickness → MBL	Systematic review + meta-analysis + TSA	Is greater thickness associated with MBL?	Association detected; heterogeneity limits causal inference.	No	Association ≠ intervention effect.	[1]
Mucosal phenotype → early/long-term MBL	Systematic review + meta-analysis	Is phenotype associated with bone loss over time?	Association differs by follow-up period.	No	Does not prove that surgical phenotype modification prevents bone loss.	[2]
SCTG/soft-tissue augmentation → MBL	Systematic review	Does augmentation alter MBL?	Inconsistent MBL effect despite more consistent soft-tissue/esthetic benefits.	Partially direct; broad augmentation evidence	Augmentation is broad; not SCTG-specific proof.	[38]
Immediate placement + SCTG	Systematic review + meta-analysis	Does CTG improve immediate implant outcomes?	Supports soft-tissue/esthetic benefits; hard-tissue outcomes are context dependent.	Partially direct; immediate-placement context	Do not extrapolate directly to delayed placement.	[49]
Timing of soft-tissue augmentation	Systematic review + meta-analysis	Does timing affect outcomes?	Timing may influence outcomes; no universally optimal strategy established.	Indirect to the SCTG → MBL question	Procedures/endpoints are not interchangeable.	[50]
CTG vs. collagen matrices	Systematic review + network meta-analysis	How do autogenous grafts compare with matrices?	CTG often favorable for soft-tissue gain; differences depend on baseline phenotype, endpoint, and time.	Comparative-material evidence; not the primary objective	Soft-tissue superiority does not prove bone preservation.	[34,35,36]
Esthetics and patient satisfaction	Systematic reviews/meta-analyses/PROM reviews	What are esthetic and patient-centered effects?	Augmentation may improve esthetic outcomes; morbidity and treatment burden matter.	No	Esthetics/PROMs are distinct from MBL.	[33,43,44,46]
Medium-/long-term stability	Systematic reviews/long-term syntheses	Are augmentation effects maintained?	Soft-tissue benefits may persist, but long-term comparisons do not show uniform SCTG superiority and bone effects remain uncertain.	Long-term context; SCTG-specific MBL inference is secondary	Do not extrapolate early benefit to long-term MBL.	[28,51,52,53]

**Table 4:** Clinical considerations for peri-implant SCTG according to clinical context, relevant assessment, therapeutic objective, and evidence context. This table summarizes the narrative synthesis and is not a validated treatment algorithm or guideline.

Evidence Context	Clinical Considerations	Therapeutic Objective	Relevant Assessment	Clinical Context
Comparative studies generally report increased soft-tissue thickness/volume; evidence for an independent long-term MBL effect remains uncertain.	Where durable autogenous soft-tissue augmentation is clinically justified, SCTG may be considered according to the treatment objective, anatomy, morbidity, and patient preference.	Increase soft-tissue thickness/volume and/or improve contour	MT/soft-tissue volume, KMW, buccal bone anatomy, implant position, esthetic context	Thin/insufficient soft tissues
Soft-tissue improvements are reported in controlled studies; hard-tissue endpoints are heterogeneous and do not establish a consistent graft-mediated MBL benefit.	Soft-tissue augmentation may be considered according to the defined soft-tissue objective; facial bone conditions should be assessed and managed independently.	Phenotype and/or contour modification	Soft-tissue phenotype, facial bone anatomy, 3D implant position, anticipated contour change	Immediate placement with thin phenotype
Comparative material studies inform material selection; they should not be interpreted as augmentation-versus-no-augmentation evidence for universal bone protection.	Where soft-tissue reconstruction is clinically indicated, autogenous or substitute-based augmentation may be considered according to defect characteristics, morbidity, and material properties.	Soft-tissue volume or contour reconstruction	Defect morphology, buccal bone dimensions, soft-tissue volume/contour	Immediate placement with small buccal dehiscence
Current evidence does not establish one universally superior timing strategy.	Timing should be individualized according to anatomy, treatment objective, prosthetic stage, and patient factors.	Soft-tissue reconstruction after tissue stabilization	Persistent volume/contour deficiency, healed anatomy, prosthetic context	Delayed/staged augmentation
The evidence identified in this focused review does not currently support routine SCTG solely for MBL preservation.	Avoid treating a favorable phenotype as an automatic indication for prophylactic grafting.	No defined soft-tissue reconstructive objective	MT/volume, KMW, STH, buccal bone anatomy, implant position, patient priorities	Favorable soft-tissue conditions without a defined reconstructive or esthetic indication
Soft-tissue and esthetic improvements are reported; successful defect correction does not establish a bone-preserving effect.	Treatment should address defect characteristics and the underlying cause; SCTG may be used for thickness/volume augmentation and selected coverage procedures.	Defect correction and soft-tissue reconstruction	Defect classification, implant position, peri-implant tissue levels, buccal bone anatomy	Existing facial soft-tissue defect
Lower MT/MT3 has been associated with mucosal-margin instability; a causal SCTG-mediated effect on long-term MBL is not established.	The cohort-derived MT3 value of 2.23 mm is a prognostic observation and should not be used as a universal surgical cutoff or generalized treatment range.	Increase MT when this is itself a clinically justified objective	Site-specific MT/MT3 measured at a defined point; assess bone independently	Low buccal MT/risk of mucosal-margin instability
Associations between limited STH and crestal remodeling have been reported; a specific SCTG → STH → long-term MBL causal pathway remains unproven.	Reported numerical values are protocol-dependent and should not be interpreted as universal treatment thresholds. Any intervention should account for implant depth and prosthetic components.	Site-specific assessment of the supracrestal compartment	STH assessed separately from MT; implant–crest relationship, implant–abutment configuration and restorative components	Limited STH/vertical supracrestal dimension

Note: This table summarizes clinical considerations arising from the focused narrative synthesis. It is not a formal guideline and does not provide a certainty-of-evidence grading. Numerical observations are retained only where directly reported by the cited studies and should not be interpreted as universal treatment thresholds. Operator experience/technique-specific competence, patient preferences, morbidity, anatomy, and prosthetic factors may modify treatment planning but are not indications by themselves.

## Data Availability

No new data were created or analyzed in this study. Data sharing is not applicable to this article.

## References

[1] Bressan E., Guazzo R., Tomasi C., Peña T.G., Moreno P.G., Caponio V.C.A., Sbricoli L., Canullo L. (2023). Influence of soft tissue thickness on marginal bone level around dental implants: A systematic review with meta-analysis and trial-sequential analysis. Clin. Oral Implant. Res..

[2] Tang P., Meng Z., Song X., Huang J., Su C., Li L. (2023). Influence of different mucosal phenotype on early and long-term marginal bone loss around implants: A systematic review and meta-analysis. Clin. Oral Investig..

[3] Puisys A., Linkevicius T. (2015). The influence of mucosal tissue thickening on crestal bone stability around bone-level implants: A prospective controlled clinical trial. Clin. Oral Implant. Res..

[4] Díaz-Sánchez M., Soto-Peñaloza D., Peñarrocha-Oltra D., Peñarrocha-Diago M. (2019). Influence of supracrestal tissue attachment thickness on radiographic bone level around dental implants: A systematic review and meta-analysis. J. Periodontal Res..

[5] Linkevicius T., Apse P., Grybauskas S., Puisys A. (2009). The influence of soft tissue thickness on crestal bone changes around implants: A 1-year prospective controlled clinical trial. Int. J. Oral Maxillofac. Implant..

[6] Terzioglu B., Guler Ayyildiz B. (2024). Effect of supracrestal tissue height on marginal bone level changes at platform-switching dental implants placed crestally and subcrestally: A randomized clinical trial. J. Dent..

[7] Tavelli L., Barootchi S., Avila-Ortiz G., Urban I.A., Giannobile W.V., Wang H.L. (2021). Peri-implant soft tissue phenotype modification and its impact on peri-implant health: A systematic review and network meta-analysis. J. Periodontol..

[8] Avila-Ortiz G., Gonzalez-Martin O., Couso-Queiruga E., Wang H.L. (2020). The peri-implant phenotype: Definition, assessment, and clinical relevance. J. Periodontol..

[9] Berglundh T., Lindhe J., Ericsson I., Marinello C.P., Liljenberg B., Thomsen P. (1991). The soft tissue barrier at implants and teeth. Clin. Oral Implant. Res..

[10] Aellos F., Clavelier A., Liu B., Wan A., Salvi G.E., Romandini M., Helms J.A. (2026). Soft-tissue integration of dental implants: Formation, maintenance, and relevance for peri-implant health. J. Periodontal Res..

[11] Sculean A., Gruber R., Bosshardt D.D. (2014). Soft tissue wound healing around teeth and dental implants. J. Clin. Periodontol..

[12] Tavelli L., Kripfgans O.D., Chan H.L., Vera Rodriguez M., Sabri H., Mancini L., Wang H.L., Giannobile W.V., Barootchi S. (2025). Doppler ultrasonographic evaluation of tissue revascularization following connective tissue graft at implant sites. J. Clin. Periodontol..

[13] Albrektsson T., Chrcanovic B., Östman P.O., Sennerby L. (2017). Initial and long-term crestal bone responses to modern dental implants. Periodontol. 2000.

[14] Davies J.E. (2003). Understanding peri-implant endosseous healing. J. Dent. Educ..

[15] Zheng Z., Ao X., Xie P., Jiang F., Chen W. (2021). The biological width around implant. J. Prosthodont. Res..

[16] Solderer A., Hicklin S.P., Aßenmacher M., Ender A., Schmidlin P.R. (2024). Influence of an allogenic collagen scaffold on implant sites with thin supracrestal tissue height: A randomized clinical trial. Clin. Oral Investig..

[17] Linkevicius T., Puisys A., Linkevicius R., Alkimavicius J., Gineviciute E., Linkeviciene L. (2020). The influence of submerged healing abutment or subcrestal implant placement on soft tissue thickness and crestal bone stability: A 2-year randomized clinical trial. Clin. Implant Dent. Relat. Res..

[18] Linkevicius T., Linkevicius R., Alkimavicius J., Linkeviciene L., Andrijauskas P., Puisys A. (2018). Influence of titanium base, lithium disilicate restoration and vertical soft tissue thickness on bone stability around triangular-shaped implants: A prospective clinical trial. Clin. Oral Implant. Res..

[19] Puisys A., Vindasiute-Narbute E., Razukevicius D., Akhondi S., Gallucci G.O., Pedrinaci I. (2024). Influence of mucosal tissue height on implant crestal bone: A 10-year follow-up of a controlled clinical trial. J. Dent..

[20] Guglielmi D., Di Domenico G.L., Aroca S., Vignoletti F., Ciaravino V., Donghia R., de Sanctis M. (2022). Soft and hard tissue changes after immediate implant placement with or without a sub-epithelial connective tissue graft: Results from a 6-month pilot randomized controlled clinical trial. J. Clin. Periodontol..

[21] Tavelli L., McGuire M.K., Zucchelli G., Rasperini G., Feinberg S.E., Wang H.L., Giannobile W.V. (2020). Extracellular matrix-based scaffolding technologies for periodontal and peri-implant soft tissue regeneration. J. Periodontol..

[22] Thoma D.S., Gasser T.J.W., Hämmerle C.H., Strauss F.J., Jung R.E. (2023). Soft tissue augmentation with a volume-stable collagen matrix or an autogenous connective tissue graft at implant sites: Five-year results of a randomized controlled trial post implant loading. J. Periodontol..

[23] Veena K.M., Shetty S.K. (2026). An analysis of the relationship between peri-implant mucosal biotype and the implant clinical outcome: A systematic review. J. Prosthet. Dent..

[24] Zuiderveld E.G., Meijer H.J.A., Gareb B., Vissink A., Raghoebar G.M. (2024). Single immediate implant placement in the maxillary aesthetic zone with and without connective tissue grafting: Results of a 5-year randomized controlled trial. J. Clin. Periodontol..

[25] Jensen S.S., Aghaloo T., Jung R.E., Bertl K., Buser D., Chappuis V., de Stavola L., Monje A., Pispero A., Roccuzzo A. (2023). Group 1 ITI Consensus Report: The role of bone dimensions and soft tissue augmentation procedures on the stability of clinical, radiographic, and patient-reported outcomes of implant treatment. Clin. Oral Implant. Res..

[26] Cairo F., Rupe C., Cavalcanti R., Landi L., Rupe A., Sforza N.M., Castelluzzo W., Di Martino M., Barbato L. (2026). Cross-Linked Volume-Stable Collagen Matrix Versus Connective Tissue Graft for Soft Tissue Augmentation at Implant Site: A Non-Inferiority, Multicenter Randomized Clinical Trial. Clin. Oral Implant. Res..

[27] Jepsen K., Jepsen S., Hämmerle C.H.F., Mancini L., Strauss F.J., Strasding M., Hicklin S., Sanz M., Sanz-Martin I., Thoma D.S. (2026). Changes in Peri-Implant Soft Tissue Dimensions Following Soft Tissue Contour Augmentation: A 3-Year Follow-Up of a Multi-Center RCT. Clin. Oral Implant. Res..

[28] Strauss F.J., Liguori M.G., Gasser T.J.W., Naenni N., Jung R.E., Thoma D.S. (2026). Soft Tissue Volume Augmentation at Single Implant Sites Applying Collagen Matrices or Connective Tissue Grafts: 10-Year Follow-Up of a Randomized Controlled Trial. J. Esthet. Restor. Dent..

[29] Hadzik J., Dominiak M. (2026). Peri-implant soft tissue thickness following connective tissue grafting or volume-stable collagen matrix augmentation: A randomized controlled trial. Dent. Med. Probl..

[30] Surdiacourt L., Christiaens V., De Bruyckere T., De Buyser S., Eghbali A., Vervaeke S., Younes F., Cosyn J. (2025). A multi-centre randomized controlled trial comparing connective tissue graft with collagen matrix to increase soft tissue thickness at the buccal aspect of single implants: 3-year results. J. Clin. Periodontol..

[31] Tavelli L., Barootchi S., Majzoub J., Chan H.L., Stefanini M., Zucchelli G., Kripfgans O.D., Wang H.L., Urban I.A. (2022). Prevalence and risk indicators of midfacial peri-implant soft tissue dehiscence at single site in the esthetic zone: A cross-sectional clinical and ultrasonographic study. J. Periodontol..

[32] Tavelli L., Sabri H., Barootchi E., Stefanini M., Zucchelli G., Wang H.L., Urban I.A., Barootchi S. (2026). Protective factors and critical mucosal thickness for the stability of the peri-implant soft tissue margin over 5 years: A longitudinal cohort study. J. Periodontol..

[33] Ramanauskaite A., Sadilina S., Schwarz F., Cafferata E.A., Strauss F.J., Thoma D.S. (2025). Soft-tissue volume augmentation during early, delayed, and late dental implant therapy: A systematic review and meta-analysis on professionally determined esthetics and self-reported patient satisfaction on esthetics. Periodontol. 2000.

[34] Tommasato G., Del Fabbro M., Oliva N., Khijmatgar S., Grusovin M.G., Sculean A., Canullo L. (2024). Autogenous graft versus collagen matrices for peri-implant soft tissue augmentation: A systematic review and network meta-analysis. Clin. Oral Investig..

[35] Cafasso E., Baima G., Campagna A., Tavelli L., Aimetti M. (2026). Soft tissue augmentation around dental implants: Techniques, timing, and comparative efficacy—A systematic review and network meta-analysis. J. Periodontal Res..

[36] Gargallo-Albiol J., Barootchi S., Tavelli L., Wang H.L. (2019). Efficacy of xenogeneic collagen matrix to augment peri-implant soft tissue thickness compared to autogenous connective tissue graft: A systematic review and meta-analysis. Int. J. Oral Maxillofac. Implant..

[37] Schmitt C.M., Brückbauer P., Schlegel K.A., Buchbender M., Adler W., Matta R.E. (2021). Volumetric soft tissue alterations in the early healing phase after peri-implant soft tissue contour augmentation with a porcine collagen matrix versus the autologous connective tissue graft: A controlled clinical trial. J. Clin. Periodontol..

[38] Fickl S., Kröger A.T., Dietrich T., Kebschull M. (2021). Influence of soft tissue augmentation procedures around dental implants on marginal bone level changes—A systematic review. Clin. Oral Implant. Res..

[39] Zuiderveld E.G., Raghoebar G.M., Vissink A., Gareb B., Meijer H.J.A. (2025). Efficacy of soft tissue augmentation in the maxillary esthetic region: A 5-year randomized controlled trial. J. Periodontol..

[40] Papantonatou L., Batas L., Delantoni A., Anagnostou E., Vouros I. (2026). Soft-Tissue Augmentation During Implant Placement in the Aesthetic Zone: Clinical, Radiographic, Histological and Patient-Reported Outcomes From a Randomised Controlled Trial Using Connective Tissue Graft and a Volume-Stable Collagen Matrix. J. Clin. Periodontol..

[41] Zucchelli G., Tavelli L., Stefanini M., Barootchi S., Mazzotti C., Gori G., Wang H.L. (2019). Classification of facial peri-implant soft tissue dehiscence/deficiencies at single implant sites in the esthetic zone. J. Periodontol..

[42] Obreja K., Ramanauskaite A., Begic A., Galarraga-Vinueza M.E., Parvini P., Schwarz F. (2021). The influence of soft-tissue volume grafting on the maintenance of peri-implant tissue health and stability. Int. J. Implant Dent..

[43] Nart J., Valles C., Vilarrasa J., Romano F., Baima G., Aimetti M. (2025). Impact of soft tissue augmentation procedures on esthetics and patient satisfaction in the treatment of peri-implant buccal soft tissue dehiscences: A systematic review and meta-analysis. Periodontol. 2000.

[44] Stefanini M., Tavelli L., Barootchi S., Sangiorgi M., Zucchelli G. (2021). Patient-reported outcome measures following soft-tissue grafting at implant sites: A systematic review. Clin. Oral Implant. Res..

[45] Arunyanak S.P., Pollini A., Ntounis A., Morton D. (2017). Clinician assessments and patient perspectives of single-tooth implant restorations in the esthetic zone of the maxilla: A systematic review. J. Prosthet. Dent..

[46] De Bruyn H., Raes S., Matthys C., Cosyn J. (2015). The current use of patient-centered/reported outcomes in implant dentistry: A systematic review. Clin. Oral Implant. Res..

[47] Zucchelli G., Sharma P., Mounssif I. (2018). Esthetics in periodontics and implantology. Periodontol. 2000.

[48] Ferrarotti F., Baima G., Mohammadi G., Carboncini C., Romano F., Aimetti M. (2025). Peri-implant soft tissue increase at small buccal bone dehiscences with either volume-stable cross-linked collagen matrix (VCMX) or connective tissue graft: A randomized controlled trial. Clin. Oral Implant. Res..

[49] Seyssens L., De Lat L., Cosyn J. (2021). Immediate implant placement with or without connective tissue graft: A systematic review and meta-analysis. J. Clin. Periodontol..

[50] Lin C.Y., Chen Z., Pan W.L., Wang H.L. (2018). Impact of timing on soft tissue augmentation during implant treatment: A systematic review and meta-analysis. Clin. Oral Implant. Res..

[51] Bienz S.P., Carrera E.R., Hüsler J., Roccuzzo A., Jung R.E., Thoma D.S. (2023). Soft tissue contour changes at implant sites with or without soft tissue grafting in the esthetic zone: A retrospective case-control study with a 12-year follow-up. Clin. Oral Implant. Res..

[52] Stefanini M., Barootchi S., Sangiorgi M., Pispero A., Grusovin M.G., Mancini L., Zucchelli G., Tavelli L. (2023). Do soft tissue augmentation techniques provide stable and favorable peri-implant conditions in the medium and long term? A systematic review. Clin. Oral Implant. Res..

[53] Tavelli L., Barootchi S., Akhondi S., Tseng E.S.C., Garcia-Valenzuela F.S., Urban I.A., Wang H.L. (2025). Long-term stability of soft tissue augmentative procedures at implant sites. Periodontol. 2000.

[54] Cosyn J., De Bruyn H., Cleymaet R. (2013). Soft tissue preservation and pink aesthetics around single immediate implant restorations: A 1-year prospective study. Clin. Implant Dent. Relat. Res..

[55] Hadzik J., Kubasiewicz-Ross P., Kujawa K., Gedrange T., Dominiak M. (2026). Methodological validation of the PIROP ultrasound-based system for measuring peri-implant soft tissue thickness in a clinical setting. J. Clin. Med..

